# An *in vitro* ileal disappearance procedure for estimating ileal digestibility of protein in weanling pigs

**DOI:** 10.5713/ab.260517

**Published:** 2026-09-01

**Authors:** Yujeong Hwang, Yoon Soo Song, Beob Gyun Kim

**Affiliations:** 1 Department of Animal Science, Konkuk University, Seoul, Korea

**Keywords:** Feed Ingredients, *In Vitro*, Protein Digestibility, Weanling Pigs

## Abstract

**Objective:**

The conventional *in vitro* nutrient digestibility procedure was developed for growing pigs whereas the procedure for weanling pigs is not available. The present study aimed to develop an *in vitro* procedure for assessing standardized ileal digestibility (SID) of crude protein (CP) in feed ingredients for weanling pigs by modifying the conventional approach used for growing pigs.

**Methods:**

In the conventional *in vitro* ileal disappearance (IVID) procedure, sample solutions added with 1 mL of pepsin (10.0 g/L) are incubated for 6.0 h in step 1 to simulate digestion in the stomach followed by adding 1 mL of pancreatin (50.0 g/L) and incubating for 18.0 h in step 2 to simulate digestion in the small intestine. To develop an IVID procedure for weanling pigs, 3 incubation periods (6.0, 4.5, and 3.0 h in step 1 and 18.0, 13.5, and 9.0 h in step 2) and 3 enzyme concentrations (pepsin 10.0, 7.5, and 5.0 g/L in step 1 and pancreatin 50.0, 37.5, and 25.0 g/L in step 2) were tested in a 3×3 factorial arrangement. Test ingredients were rice, corn, wheat, rapeseed meal, soybean meal, fermented soybean meal, and soy protein concentrate.

**Results:**

The IVID of CP, measured using the conventional procedure, exhibited an average of 5.0% deviation from the *in vivo* SID of CP in weanling pigs. Based on the quadratic response surface analysis, the use of 1 mL of pepsin at 7.6 g/L with the incubation period of 4.4 h in step 1 followed by adding 1 mL of pancreatin at 38.1 g/L with the incubation period of 13.3 h in step 2 minimized the absolute difference between IVID of CP and *in vivo* SID of CP to 3.6% on average for weanling pigs.

**Conclusion:**

The *in vitro* procedure for weanling pigs was refined by reducing the incubation periods and enzyme concentrations to more accurately assess the ileal digestibility of protein in feed ingredients compared with the conventional *in vitro* procedure.

## INTRODUCTION

Digestibility of crude protein (CP) and amino acids is a key parameter for evaluating the nutritive value of feed ingredients for pigs [1,2]. Animal experiments are regarded as reliable methods for assessing the nutritional values of feed ingredients fed to pigs. As an alternative to *in vivo* trials, *in vitro* digestion assays have been employed to evaluate swine feeds [3–5] based on the high correlation between the *in vitro* ileal disappearance (IVID) of CP and ileal protein digestibility in pigs obtained from *in vivo* assays [6].

The conventional 2-step *in vitro* procedure was developed to estimate the ileal protein digestibility by mimicking digestion and absorption processes of growing pigs ranging from 40 to 60 kg body weight [6]. However, the *in vitro* assays may not accurately estimate the standardized ileal digestibility (SID) of CP in weanling pigs which have relatively underdeveloped digestive and absorptive capacities compared to growing pigs [7–9]. The ileal digestibility of CP in weanling pigs has been reported to be less than that in growing pigs [10,11]. Therefore, the conventional *in vitro* procedure developed for growing pigs may overestimate the CP digestibility in feeds fed to weanling pigs. The present experiment aimed to develop an *in vitro* procedure for estimating the SID of CP in feed ingredients for weanling pigs by modifying the conventional procedure.

## MATERIALS AND METHODS

### Feed ingredients and experimental design

To develop an IVID procedure for estimating the SID of CP in weanling pigs, rice, corn, wheat, rapeseed meal (RSM), soybean meal (SBM), fermented SBM (FSBM), and soy protein concentrate (SPC) were employed (Table 1). In the 2-step *in vitro* assays, 3 incubation periods (6.0, 4.5, and 3.0 h in step 1 and 18.0, 13.5, and 9.0 h in step 2) and 3 enzyme concentrations (pepsin 10.0, 7.5, and 5.0 g/L in step 1 and pancreatin 50.0, 37.5, and 25.0 g/L in step 2) were tested in a 3×3 factorial arrangement. The tested incubation periods and enzyme concentrations corresponded to 100%, 75%, and 50% of the incubation periods and enzyme concentrations used in the conventional 2-step *in vitro* procedure, respectively [6]. The reduction ratio of incubation period or enzyme concentration was kept constant in both steps 1 and 2.

### An *in vitro* assay to develop a procedure for weanling pigs

The samples were ground to less than 1 mm before *in vitro* procedures (Cyclotec 1093; Foss Tecator AB). Two-step *in vitro* assays were conducted to determine the IVID of dry matter (DM) and CP in 7 test ingredients based on the method described by Boisen and Fernández [6]. In the first step, 1 g of an ingredient sample was transferred into a 100-mL conical flask and then 25 mL of sodium phosphate buffer solution (0.1 *M*, pH 6.0) and 10 mL of HCl (0.2 *M*, pH 0.7) were added to the flask. To simulate digestion conditions in the stomach of pigs, the pH was adjusted to 2.0 using 1 *M* HCl or 1 *M* NaOH, and 1 mL of freshly prepared pepsin solution at 10.0, 7.5, or 5.0 g/L (≥250 units/mg solid, P7000, Pepsin from porcine gastric mucosa; Sigma-Aldrich) was added to the flask. To avoid bacterial fermentation, 0.5 mL of chloramphenicol (C0378, chloramphenicol; Sigma-Aldrich) solution (5 g/L ethanol) was also added. The flasks were closed with a silicone stopper and incubated in a shaking incubator (LSI-3016R; Daihan Labtech) at 39°C and 125 rpm for 6.0, 4.5, or 3.0 h as the final procedure of the first step. In the second step, 10 mL of sodium phosphate buffer solution (0.2 *M*, pH 6.8) and 5 mL of NaOH (0.6 *M*, pH 13.8) were added to the flasks. Then, the pH was adjusted to 6.8 using 1 *M* HCl or 1 *M* NaOH, and 1 mL of freshly prepared pancreatin solution at 50.0, 37.5, or 25.0 g/L (4×USP, P1750, Pancreatin from porcine pancreas; Sigma-Aldrich) was added. Pancreatin solution was centrifuged at 2,468×g for 2 min and only supernatant was used. Thereafter, the flasks were incubated in the shaking incubator (LSI-3016R; Daihan Labtech) at 39°C and 125 rpm for 18.0, 13.5, or 9.0 h.

Following the incubation, 5 mL of 20% sulfosalicylic acid solution was added and the flasks were left for 30 min at room temperature to precipitate the indigestible protein. After precipitation, undigested samples were filtered through pre-dried and pre-weighed glass filter crucibles (Filter Crucibles CFE Por. 2; Robu) containing 500 mg of acid-insoluble ash (Celite 545; Daejung) which helps to inhibit plugging the filter. The flasks were rinsed twice with 1% sulfosalicylic acid solution, and 10 mL of 95.0% ethanol and 99.5% acetone were added twice to the glass filter crucibles. The glass filter crucibles with undigested samples were dried at 80°C for 24 h. After cooling in a desiccator for 1 h, glass filter crucibles were weighed to calculate the IVID of DM in the test ingredients. The undigested residues in filter crucibles were collected for analyzing CP concentration to calculate the IVID of CP. The values for blanks were estimated to correct the DM and CP contents in the residues that did not originate from the feed ingredients based on the amounts of pepsin and pancreatin added according to Song et al. [12].

### Chemical analyses

All chemical analyses were performed according to the AOAC International [13]. The feed ingredients and *in vitro* undigested residues were analyzed for DM (method 930.15) and CP (method 976.05). Gross energy (Parr 6400; Parr Instruments), ether extract (method 920.39), ash (method 942.05), amylase-treated neutral detergent fiber inclusive of residual ash (method 2002.04), and acid detergent fiber inclusive of residual ash (method 973.18) in feed ingredients were also analyzed.

### Calculations

The IVID of DM was calculated using the following equation:


(1) $$\text{IVID of DM }(\%)=({\text{DM}}_{\text{ingredient}}-{\text{DM}}_{\text{residue}}+{\text{DM}}_{\text{blank}})÷{\text{DM}}_{\text{ingredient}}\times 100\%$$

where DM_ingredient_ (g) is the amount of test ingredient DM, DM_residue_ (g) is the amount of DM residues after *in vitro* digestion procedures, and DM_blank_ (g) is the amount of DM residues in the blank estimated according to Song et al. [12].

After the 2-step *in vitro* assays, the undigested residues of test ingredient and Celite were collected, weighed, and analyzed for CP. The IVID of CP was calculated using the following equation:


(2) $$\begin{array}{l} \text{IVID of CP }(\%)=\\ ([{\text{DM}}_{\text{ingredient}}\times {\text{CP}}_{\text{ingredient}}]-[{\text{DM}}_{\text{residue}}\times {\text{CP}}_{\text{residue}}]+\\ {}[{\text{DM}}_{\text{blank}}\times {\text{CP}}_{\text{blank}}])÷({\text{DM}}_{\text{ingredient}}\times {\text{CP}}_{\text{ingredient}})\times 100\% \end{array}$$

where CP_ingredient_ and CP_residue_ are the CP concentrations (g/kg) expressed as DM basis in the test ingredient and the undigested residues, respectively, and CP_blank_ is the amount of CP residues in the blank estimated according to Song et al. [12].

### Data collection for standardized ileal digestibility of crude protein in weanling pigs

To develop a 2-step *in vitro* procedure for estimating the SID of CP in weanling pigs, peer-reviewed research articles were obtained. Multiple searches were performed in April 2026 to find publications related to evaluation of SID of CP in feed ingredients fed to weanling pigs. The databases used in the literature search included PubMed and Google Scholar, using the keywords rice, corn, wheat, RSM, SBM, FSBM, SPC, ileal digestibility, and weanling pigs. The search included publications from 2,000 onwards and excluded results from patents and publications not in English. After obtaining all relevant publications, strict selection criteria were applied to ensure the inclusion of only appropriate experiments. Studies that used rice, corn, wheat, RSM, SBM, FSBM, or SPC as the sole source of nitrogen in a corn starch-based diet to determine the SID of CP in the feed ingredients were exclusively included in the dataset. Finally, a total of 23 data for SID of CP in rice (n = 1), corn (n = 2), wheat (n = 2), RSM (n = 1), SBM (n = 7), FSBM (n = 7), and SPC (n = 3) fed to weanling pigs were obtained from 12 research articles [1,14–24].

### Statistical analyses

Experimental data were analyzed using the GLM procedure of SAS [25]. Preplanned orthogonal polynomial contrasts were employed to analyze linear and quadratic effects of incubation periods and enzyme concentrations, and their interaction in IVID of DM and CP. The absolute differences between the IVID of CP and the SID of CP were calculated for all combinations of incubation periods and enzyme concentrations. In addition, the NLIN procedure [26] was used to determine the optimal incubation periods and enzyme concentrations for developing the 2-step *in vitro* procedure for estimating the SID of CP in weanling pigs. A quadratic response surface model was fitted employing the least squares method with incubation period and enzyme concentration as independent variables and the absolute difference between the IVID and SID of CP as the dependent variable. To visualize the fitted model and the estimated minimum point, 3-dimensional surface plots were generated using the G3D procedure. An individual flask was the experimental unit and an alpha level used for determining statistical significance was declared at 0.05.

## RESULTS

As the incubation period was reduced, the IVID of DM linearly decreased (p<0.001) in rice, wheat, and RSM and quadratically decreased (p = 0.012) in SBM (Table 2). As the enzyme concentration was reduced, the IVID of DM linearly decreased (p<0.05) in wheat, RSM, and SBM and quadratically decreased (p = 0.045) in rice. For corn, FSBM, and SPC, the magnitude of the decrease in IVID of DM with the reduction of enzyme concentration increased with reducing incubation period (linear interaction p<0.05).

The IVID of CP in rice, RSM, and FSBM linearly decreased (p<0.001) with reducing incubation period (Table 3). The IVID of CP in corn, wheat, and SPC showed a quadratic response (p<0.05) with reducing incubation period. As the enzyme concentration reduced, the IVID of CP linearly decreased (p<0.001) in corn, wheat, RSM, and SPC and quadratically decreased (p<0.01) in rice and FSBM. For SBM, the magnitude of the decrease in IVID of CP with the reduction of enzyme concentration increased with reducing incubation period (linear interaction p<0.001).

The IVID of CP measured using the conventional procedure (pepsin 10.0 g/L for 6.0 h in step 1 and pancreatin 50.0 g/L for 18.0 h in step 2) deviated from the weanling pig’s SID of CP by 5.0% on average (Table 4). When incubation periods and enzyme concentrations were reduced to 75% of the conventional procedure, the IVID of CP deviated from the SID of CP ranged from 0.5% to 6.7% (mean = 3.6%). A quadratic response surface analysis indicated that the optimal *in vitro* procedure for weanling pigs for minimizing the absolute difference between IVID and SID of CP calculated in Table 4 was to use 1 mL of pepsin at 7.6 g/L with the incubation period of 4.4 h in step 1 followed by the addition of 1 mL of pancreatin at 38.1 g/L and incubation for 13.3 h in step 2 (Figure 1).

## DISCUSSION

A conventional 2-step *in vitro* procedure developed by Boisen and Fernández [6] has been adopted for estimating CP digestibility in feed ingredients and diets for pigs [3–5]. However, the conventional *in vitro* procedure may not be applicable for weanling pigs because the body weight of pigs was 40 to 60 kg for developing the *in vitro* procedure [6], and weanling pigs have less digestive capability compared with growing pigs [8,10,27]. According to the meta-analysis by Pedersen et al. [11], the SID of CP in dehulled SBM was less in pigs less than 20 kg body weight compared with heavier pigs likely due to the relatively immature digestive system of weanling pigs. Considering the low digestive capacity of weanling pigs, Son and Kim [5] reduced incubation periods and enzyme concentrations to 50% of the conventional 2-step *in vitro* assays for determining IVID of CP in soybean ingredients for weanling pigs. However, studies on titrating the incubation periods and the enzyme concentrations are lacking for the 2-step *in vitro* procedure for weanling pigs. Therefore, the present experiment aimed to develop a 2-step *in vitro* procedure for estimating the SID of CP for weanling pigs by testing various incubation periods and enzyme concentrations.

The analyzed concentrations of DM, CP, ash, and amylase-treated neutral detergent fiber in 7 feed ingredients used in the present experiment were close to the values from the literature [19,24,28,29]. In addition, the concentrations of CP and amylase-treated neutral detergent fiber in 7 feed ingredients used in the present experiment were similar to, or within the range of, the composition of each ingredient reported in the studies from which the SID of CP data were obtained. The IVID of CP in feed ingredients determined in the present experiment using the conventional 2-step *in vitro* assay (pepsin 10.0 g/L for 6.0 h in step 1 and pancreatin 50.0 g/L for 18.0 h in step 2) was comparable to the observations in previous studies [30–33].

The interaction between incubation periods and enzyme concentrations on the IVID of CP in SBM observed in the present experiment indicates that the IVID of CP in SBM decreased by the enzyme concentration reductions more sharply when the incubation periods were shortened. Pepsin is a type of protease, and pancreatin contains protease, amylase, trypsin, lipase, and ribonuclease [12]. The activities of the enzymes are affected by several factors such as enzyme concentrations, digestion inhibitors in feed ingredients, and incubation conditions including temperature, pH, and incubation periods [2]. Therefore, the interaction between incubation periods and enzyme concentrations in IVID of CP in SBM is likely due to the reduced degradation of large-molecule proteins in SBM both by lowered incubation periods and enzyme concentrations [34,35].

In contrast to SBM, the lack of interaction between incubation periods and enzyme concentrations observed in FSBM is likely due to smaller molecular size and less anti-nutritional factors [34]. Fermented SBM used in the present experiment was produced by *Bacillus subtilis* fermentation which hydrolyzes anti-nutritional factors and degrades large protein molecules into smaller peptides [11,24], making FSBM less susceptible to restricted digestion conditions.

In the case of SPC, although the interaction between incubation periods and enzyme concentrations was not observed, the IVID of CP was more sharply decreased by reducing enzyme concentrations compared with SBM or FSBM. In addition, the decrease of IVID of CP by reduced enzyme was independent of incubation period. Although the reason for this observation remains unclear, the high concentration of insoluble carbohydrates in SPC may partially explain the present results [24]. Soy protein concentrate is produced by aqueous alcohol leaching SBM to fortify protein concentration by removing soluble carbohydrate fractions [22,36]. The insoluble carbohydrates concentrated in SPC during the leaching process potentially lower nutrient digestibility and increase sensitivity to the reduced enzymes in the *in vitro* procedures. Furthermore, the quantity of substrate protein relative to protease added during the *in vitro* procedure is also decisive for enzyme activity. There were more protein contents in 1 g of SPC than in 1 g of SBM, and thus, the quantity of protein in SPC may have exceeded the limit of digestion by the protease. Therefore, the effects of lowering enzyme concentrations may be more critical for SPC independent of incubation period compared with SBM and FSBM.

The varying magnitude of the changes of the differences between IVID and SID of CP depending on incubation periods and enzyme concentrations among feed ingredients may be attributed to differences in the physicochemical properties of feed ingredients. The variation in the characteristics of feed ingredients can result in different digestion processes depending on whether the condition is *in vivo* or *in vitro*. Boisen and Eggum [2] suggested that fiber and anti-nutritional factors such as trypsin inhibitors and glucosinolates could cause a much smaller effect on *in vitro* trials compared with *in vivo* conditions due to the specific and quantitative action of these anti-nutritional factors particularly under *in vivo* conditions. For example, RSM used showed the greatest difference (8.9%) between the IVID of CP under the conventional *in vitro* procedure and the SID of CP determined in weanling pigs, which is likely because glucosinolate and fiber contents in RSM may have negatively affected *in vivo* nutrient digestibility, but not much under the *in vitro* digestion conditions [2]. An optimal 2-step *in vitro* procedure for the least difference between IVID and SID of CP can be developed for each feed ingredient based on the present results. In the present work, however, a single robust procedure was suggested to apply various ingredients to avoid complexity of the procedures. The optimal IVID procedure for weanling pigs based on the quadratic response surface analysis is very close to 75% of incubation periods and enzyme concentrations in the conventional IVID procedure. Consistently, the difference between the IVID and SID of CP was the least (mean = 3.55%) when incubation periods and enzyme concentrations were reduced to 75% (pepsin 7.5 g/L for 4.5 h in step 1 and pancreatin 37.5 g/L for 13.5 h in step 2) of the conventional procedure. This difference supports the minimal Y-value of 3.66% which indicates the absolute difference between the IVID of CP and SID of CP in the 3-dimensional quadratic response surface analysis. The minimal Y-value indicates the expected error when the IVID procedure suggested in the present work is employed for estimating SID of CP in ingredients fed to weanling pigs. Considering that the IVID of CP measured using the conventional procedure deviated from the SID of CP for weanling pigs by 5.00% on average, the IVID procedure for weanling pigs developed by lowering the incubation period and the enzyme concentration to 74.1% and 76.2%, respectively, of the conventional IVID procedure increased the accuracy of estimating SID of CP by 1.3 percentage units.

Although the newly developed IVID procedure suggested in the present experiment can be used for estimating protein digestibility of ingredients for weanling pigs, the information on the availability of amino acids is necessary in practical diet formulations. Nevertheless, the proposed IVID procedure can be used as a preliminary tool for screening feed ingredients for weanling pigs. The development of an *in vitro* procedure for estimating the SID of amino acids in weanling pigs should be warranted in future studies.

In addition, the newly developed procedure in the present work has not yet been validated. Three main criteria were considered when selecting literature data on the SID of CP in weanling pigs for the procedure modification: first, digestibility was measured in weanling pigs; second, the SID of CP rather than apparent ileal digestibility was reported; and third, the chemical composition of feed ingredients was similar to the composition of those used in the present experiment. Due to these criteria, the unequal numbers of data for the ingredients were inevitable, which potentially cause biases in the present work. All available data on the SID of CP in feed ingredients for weanling pigs were used to modify the procedure. Future research is required to validate the newly developed procedure in the present work.

## CONCLUSION

Reducing the incubation period and the enzyme concentration to 74.1% and 76.2%, respectively, of the conventional *in vitro* procedure minimized the absolute difference between *in vitro* ileal protein disappearance and *in vivo* standardized ileal protein digestibility for weanling pigs based on the 3-dimensional quadratic response surface analysis. The newly developed *in vitro* procedure can be used for estimating ileal digestibility of protein in various feed ingredients for weanling pigs.

## Figures and Tables

**Figure 1:**
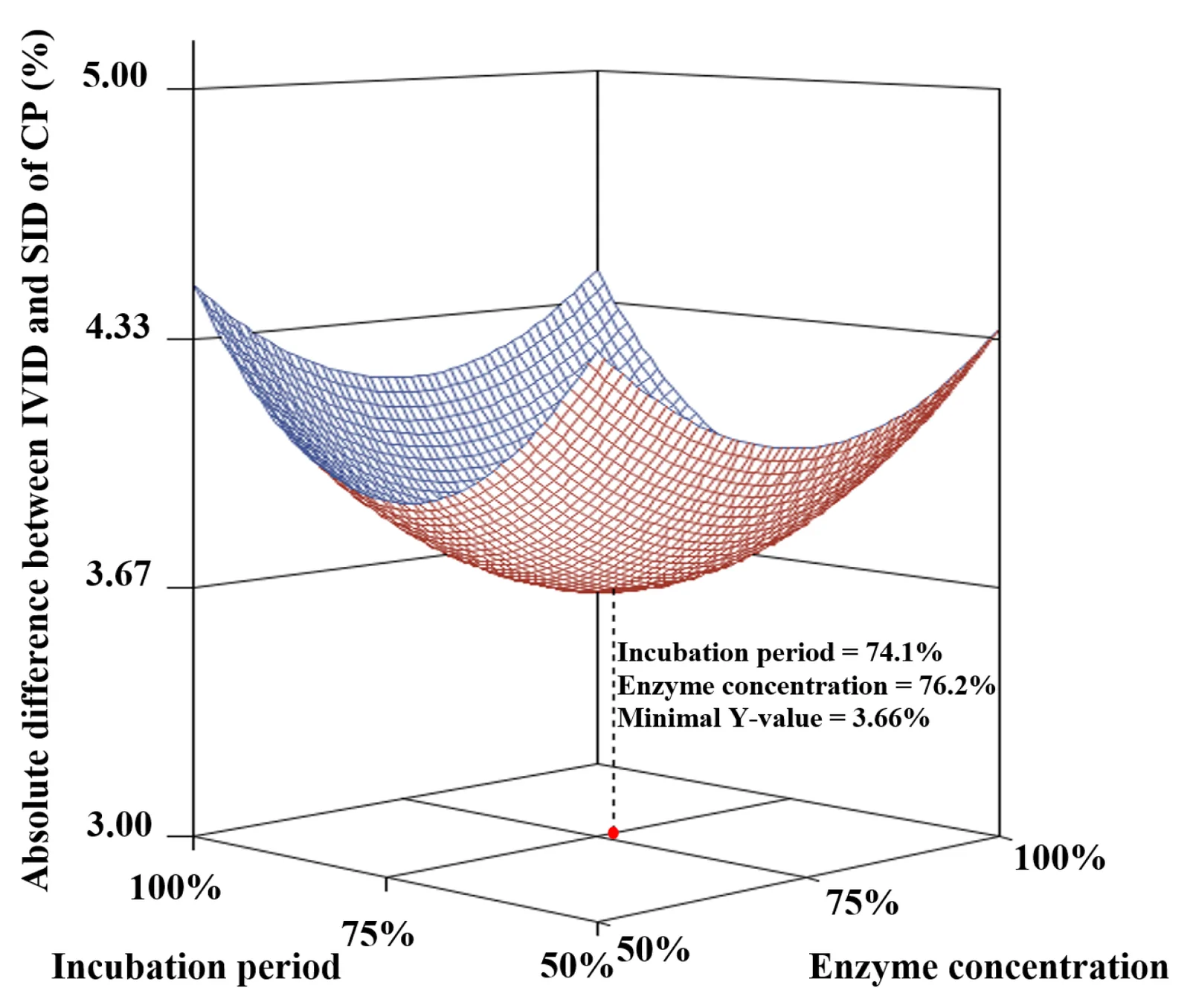
A quadratic response surface analysis of the absolute difference between the *in vitro* ileal digestibility (IVID, %) and standardized ileal digestibility (SID, %) of crude protein (CP) derived from 12 research articles for weanling pigs according to incubation period and enzyme concentration in the 2-step *in vitro* procedure. A quadratic model indicated that the minimum absolute difference was obtained at an incubation period of 74.1% and an enzyme concentration of 76.2%, with the following equation: Y = 3.66+0.000763× (X_1_–74.1)^2^+0.000448×(X_2_–76.2)^2^ where X_1_ is the incubation period and X_2_ is the enzyme concentration. On the incubation period axis, 100%, 75%, and 50% correspond to 6.0, 4.5, and 3.0 h in step 1, and 18.0, 13.5, and 9.0 h in step 2, respectively. On the enzyme concentration axis, 100%, 75%, and 50% correspond to pepsin concentrations of 10.0, 7.5, and 5.0 g/L in step 1, and pancreatin concentrations of 50.0, 37.5, and 25.0 g/L in step 2, respectively.

**Table 1:** Analyzed composition of test ingredients (as-is basis)

Item	Rice	Corn	Wheat	RSM	SBM	FSBM	SPC
Gross energy (kcal/kg)	3,610	3,868	3,890	4,281	4,119	4,455	4,400
Dry matter (%)	87.0	86.7	90.5	89.6	88.4	93.4	91.2
Crude protein (%)	7.5	9.3	11.6	34.5	45.9	52.6	58.4
Ether extract (%)	0.3	3.8	3.3	2.3	1.9	0.9	0.4
Ash (%)	0.8	1.9	2.7	8.4	7.2	7.4	7.1
Amylase-treated neutral detergent fiber (%)	0.8	8.5	9.2	28.7	7.1	3.5	9.8
Acid detergent fiber (%)	0.4	2.1	2.6	17.7	3.0	1.9	3.9

All values were determined in duplicate.

RSM, rapeseed meal; SBM, soybean meal; FSBM, fermented SBM; SPC, soy protein concentrate.

**Table 2:** Effects of incubation periods (IP) and enzyme concentrations (EC) on the *in vitro* ileal disappearance (%) of dry matter in test ingredients (n = 3)

Item				Rice	Corn	Wheat	RSM	SBM	FSBM	SPC
IP (h)		EC (g/L)								
Step 1	Step 2	Step 1	Step 2							
6.0	18.0	10.0	50.0	87.2	86.1	87.9	62.6	77.2	76.0	71.5
6.0	18.0	7.5	37.5	86.5	84.9	87.5	62.2	76.4	75.1	71.9
6.0	18.0	5.0	25.0	85.9	83.0	86.6	61.7	74.3	73.3	68.0
4.5	13.5	10.0	50.0	85.0	84.2	86.9	61.9	75.8	75.1	68.8
4.5	13.5	7.5	37.5	84.0	82.5	86.1	61.7	74.5	72.7	67.5
4.5	13.5	5.0	25.0	79.5	78.8	84.1	60.8	74.9	70.8	63.6
3.0	9.0	10.0	50.0	79.2	77.5	85.0	60.4	73.7	74.1	66.3
3.0	9.0	7.5	37.5	80.1	75.9	83.3	59.1	72.1	72.2	65.4
3.0	9.0	5.0	25.0	76.7	69.6	82.2	58.9	69.8	67.4	55.4
SEM				0.8	0.8	0.5	0.6	0.6	0.9	0.6
p-values for contrast			L_IP_	<0.001	<0.001	<0.001	<0.001	<0.001	<0.001	<0.001
			Q_IP_	0.703	0.001	0.429	0.139	0.012	0.812	0.649
			L_EC_	<0.001	<0.001	<0.001	0.023	<0.001	<0.001	<0.001
			Q_EC_	0.045	0.053	0.573	0.900	0.909	0.409	<0.001
			L_IP_×L_EC_	0.485	0.010	0.127	0.651	0.432	0.038	<0.001
			L_IP_×Q_EC_	0.127	0.208	0.453	0.547	0.767	0.513	0.037
			Q_IP_×L_EC_	0.022	0.922	0.386	0.890	0.021	0.819	0.066
			Q_IP_×Q_EC_	0.568	0.782	0.409	0.499	0.160	0.363	0.036

RSM, rapeseed meal; SBM, soybean meal; FSBM, fermented SBM; SPC, soy protein concentrate; SEM, standard error of the mean; L_IP_, linear effect of IP; Q_IP_, quadratic effect of IP; L_EC_, linear effect of EC; Q_EC_, quadratic effect of EC.

**Table 3:** Standardized ileal digestibility (SID, %) of crude protein (CP) for weanling pigs in the literature and effects of incubation periods (IP) and enzyme concentrations (EC) on the *in vitro* ileal disappearance (IVID, %) of CP in test ingredients (n = 3)

Item				Rice	Corn	Wheat	RSM	SBM	FSBM	SPC
SID of CP for weanling pigs1)				93.7	77.5	80.9	78.0	85.2	86.2	84.9
IVID of CP										
IP (h)		EC (g/L)								
Step 1	Step 2	Step 1	Step 2							
6.0	18.0	10.0	50.0	92.4	78.7	86.3	86.9	90.6	91.4	92.4
6.0	18.0	7.5	37.5	92.2	76.1	84.2	86.3	90.4	90.6	90.1
6.0	18.0	5.0	25.0	89.5	71.8	83.7	86.0	89.1	88.2	85.7
4.5	13.5	10.0	50.0	89.3	75.5	83.9	85.9	90.6	89.4	87.2
4.5	13.5	7.5	37.5	89.0	74.8	84.3	84.7	88.9	89.3	85.5
4.5	13.5	5.0	25.0	85.7	71.5	83.2	81.6	87.8	86.3	79.8
3.0	9.0	10.0	50.0	86.1	71.4	81.3	85.1	86.8	88.6	84.4
3.0	9.0	7.5	37.5	85.9	67.2	80.8	84.1	86.7	87.6	82.7
3.0	9.0	5.0	25.0	83.8	66.3	79.7	81.4	82.5	85.1	79.3
SEM				0.5	1.1	0.5	0.8	0.3	0.5	1.1
p-values for contrast			L_IP_	<0.001	<0.001	<0.001	<0.001	<0.001	<0.001	<0.001
			Q_IP_	0.354	0.015	0.003	0.131	<0.001	0.479	0.045
			L_EC_	<0.001	<0.001	<0.001	<0.001	<0.001	<0.001	<0.001
			Q_EC_	0.002	0.845	0.832	0.353	0.001	0.009	0.106
			L_IP_×L_EC_	0.561	0.409	0.331	0.113	<0.001	0.792	0.458
			L_IP_×Q_EC_	0.760	0.183	0.241	0.446	0.005	0.839	0.897
			Q_IP_×L_EC_	0.320	0.313	0.108	0.168	0.934	0.723	0.443
			Q_IP_×Q_EC_	0.648	0.295	0.190	0.642	0.001	0.365	0.502

1) A total of 23 data for SID of CP in rice, corn, wheat, RSM, SBM, FSBM, and SPC fed to weanling pigs were obtained from 12 research articles [1,14–24]. The average initial body weights of pigs reported in the articles for rice (n = 1), corn (n = 2), wheat (n = 2), RSM (n = 1), SBM (n = 7), FSBM (n = 7), and SPC (n = 3) were 14.1, 12.3, 10.6, 14.1, 10.5, 9.6, and 10.9 kg, respectively.

RSM, rapeseed meal; SBM, soybean meal; FSBM, fermented SBM; SPC, soy protein concentrate; SEM, standard error of the mean; L_IP_, linear effect of IP; Q_IP_, quadratic effect of IP; L_EC_, linear effect of EC; Q_EC_, quadratic effect of EC.

**Table 4:** The differences between the *in vitro* ileal disappearance (IVID, %) of crude protein (CP) with varying incubation periods (IP) and enzyme concentrations (EC) and standardized ileal digestibility (SID, %) of CP derived from 12 research articles for weanling pigs

Item				IVID of CP minus SID of CP						

				Rice	Corn	Wheat	RSM	SBM	FSBM	SPC
IP (h)		EC (g/L)								
Step 1	Step 2	Step 1	Step 2							
6.0	18.0	10.0	50.0	−1.3	1.2	5.4	8.9	5.4	5.2	7.5
6.0	18.0	7.5	37.5	−1.5	−1.3	3.4	8.3	5.2	4.5	5.1
6.0	18.0	5.0	25.0	−4.2	−5.6	2.8	8.0	3.9	2.0	0.8
4.5	13.5	10.0	50.0	−4.4	−1.9	3.1	7.9	5.4	3.2	2.2
4.5	13.5	7.5	37.5	−4.7	−2.7	3.5	6.7	3.6	3.2	0.5
4.5	13.5	5.0	25.0	−8.0	−6.0	2.4	3.6	2.6	0.2	−5.1
3.0	9.0	10.0	50.0	−7.6	−6.1	0.5	7.1	1.5	2.5	−0.5
3.0	9.0	7.5	37.5	−7.8	−10.3	−0.1	6.1	1.5	1.4	−2.3
3.0	9.0	5.0	25.0	−9.9	−11.1	−1.1	3.4	−2.7	−1.0	−5.6

RSM, rapeseed meal; SBM, soybean meal; FSBM, fermented SBM; SPC, soy protein concentrate.

## Data Availability

Upon reasonable request, the datasets of this study can be available from the corresponding author.
